# Cellular characterization of ultrasound-stimulated microbubble radiation enhancement in a prostate cancer xenograft model

**DOI:** 10.1242/dmm.012922

**Published:** 2014-01-30

**Authors:** Azza A. Al-Mahrouki, Sara Iradji, William Tyler Tran, Gregory J. Czarnota

**Affiliations:** 1 Physical Sciences, Sunnybrook Research Institute, Sunnybrook Health Sciences Centre, 2075 Bayview Avenue, Toronto, ON M4N 3M5, Canada.; 2 Department of Radiation Oncology, Sunnybrook Health Sciences Centre, 2075 Bayview Avenue, Toronto, ON M4N 3M5, Canada.; 3 Department of Radiation Oncology, Faculty of Medicine, University of Toronto, Toronto, ON M5S 3E2, Canada.; 4 Department of Medical Biophysics, Faculty of Medicine, University of Toronto, Toronto, ON M5S 3E2, Canada.

**Keywords:** Angiogenesis, Microbubbles, Proliferation, Radiation, Ultrasound

## Abstract

Tumor radiation resistance poses a major obstacle in achieving an optimal outcome in radiation therapy. In the current study, we characterize a novel therapeutic approach that combines ultrasound-driven microbubbles with radiation to increase treatment responses in a prostate cancer xenograft model in mice. Tumor response to ultrasound-driven microbubbles and radiation was assessed 24 hours after treatment, which consisted of radiation treatments alone (2 Gy or 8 Gy) or ultrasound-stimulated microbubbles only, or a combination of radiation and ultrasound-stimulated microbubbles. Immunohistochemical analysis using *in situ* end labeling (ISEL) and terminal deoxynucleotidyl transferase dUTP nick-end labeling (TUNEL) revealed increased cell death within tumors exposed to combined treatments compared with untreated tumors or tumors exposed to radiation alone. Several biomarkers were investigated to evaluate cell proliferation (Ki67), blood leakage (factor VIII), angiogenesis (cluster of differentiation molecule CD31), ceramide-formation, angiogenesis signaling [vascular endothelial growth factor (VEGF)], oxygen limitation (prolyl hydroxylase PHD2) and DNA damage/repair (γH2AX). Results demonstrated reduced vascularity due to vascular disruption by ultrasound-stimulated microbubbles, increased ceramide production and increased DNA damage of tumor cells, despite decreased tumor oxygenation with significantly less proliferating cells in the combined treatments. This combined approach could be a feasible option as a novel enhancing approach in radiation therapy.

## INTRODUCTION

Radiotherapy is considered to be an effective conventional therapy for various tumors (Folkman and Camphausen, 2001; Spiegelhalter et al., 2011; Wei et al., 2012). It is used as a primary modality for the treatment of early-stage cancers of the head and neck, breast, prostate, cervix and some lung and skin cancers, as well as more advanced tumors (Becher et al., 2010; Huang et al., 2010; Leung and Ngan, 2010; Connell and Hellman, 2009; Pchejetski et al., 2010). The action of ionizing radiation causes damage to cellular DNA in both tumor cells and normal tissue. Cell death from ionizing radiation exposure is primarily due to abnormalities within repair mechanisms in tumor cells. Radiation effects on normal cells, which have better repair mechanisms, have the potential of forming long-term damage and secondary cancers (Bastianutto et al., 2007). Current strategies in radiation oncology are implementing modified fractionation schemes and high-precision dose delivery methods to avoid normal tissues to minimize radiation effects. However, biological obstacles currently stand in the way of achieving an optimal therapeutic ratio. Radiation resistance is a challenge in many treatments (Wei et al., 2012; Diepart et al., 2012; Garden et al., 1991; Bao et al., 2006) in part owing to factors such as hypoxia, genetic instability, tumor hypervascularity and the presence of tumor stem cells (Diehn et al., 2009). These cellular behaviors contribute to a spectrum of obstacles in cancer therapy. Other challenges such as adaptive pathways and cellular migration through the vascular or the lymphatic systems (Mantovani et al., 2008; Bos et al., 2009) can add to the complexity of developing an effective therapy. Radiation-sensitizing agents are mostly systemic agents that have associated toxicities, limiting their use.

Radiation-enhancing agents span chemical agents including chemotherapy drugs, which enhance radiation effects, to hypoxic cell sensitizers, and also include biophysical modalities such as hyperthermia. We have recently demonstrated the novel efficacy of ultrasound-driven microbubbles to act as effective radiation-enhancing agents causing cell stress and disruption of endothelial cells within the tumor vasculature (Czarnota et al., 2012). Ultrasound waves stimulate microbubbles by causing them to oscillate, expand and then collapse, resulting in mechanical and physical changes in the surrounding environment, and can be used in cancer therapy either as vehicles for targeted drug delivery (Ibsen et al., 2013) or in combination with radiotherapy. Using bubble therapy with radiation causes endothelial cell death and vascular disruption with supra-additive effects *in vivo*, as demonstrated in prostate and bladder xenograft tumor models (Czarnota et al., 2012; Tran et al., 2012). In that work, combining 2 or 8 Gy doses of radiation with ultrasound-stimulated microbubble treatments (each on their own resulted in minimal cell death) caused 40–60% tumor cell death within 24 hours. Multiple treatments of tumors with ultrasound-stimulated microbubbles combined with radiation in which bubbles were burst also resulted in superior animal survival compared with treatments with either modality alone (Czarnota et al., 2012).

Microbubbles are lipid-coated microspheres that encapsulate an inert gas. Currently, perfluoropropane microbubbles are being used in sonography as contrast agents for clinical imaging and diagnostic purposes (Badea et al., 2009; Cosgrove, 2006). Recent advances have investigated targeted approaches by conjugating antibodies to microbubble surfaces in order to enhance specificity to the vasculature, leading to improved diagnostic and therapeutic monitoring (Korpanty et al., 2007). Additionally, microbubbles can be used as therapeutic vehicles and have facilitated and improved bimolecular, drug and gene delivery by enhancing cellular permeability (Karshafian et al., 2009; Hernot and Klibanov, 2008; Phillips et al., 2010). Haag et al. reported the successful delivery of antisense oligo-DNA against human androgen receptor (Haag et al., 2006). When combined with radiation, such microbubble agents seem to stimulate a ceramide-dependent cell death pathway in endothelial cells (Al-Mahrouki et al., 2012; Czarnota et al., 2012). This leads to endothelial cell apoptosis and rapid onset of cell death within 24 hours when such ultrasound-stimulated microbubble treatments are combined with 2 Gy or higher-dose radiation treatments (Czarnota et al., 2012).

**TRANSLATIONAL IMPACT**

**Clinical issue**

Resistance to radiotherapy is a challenge in the treatment of many types of cancer. In addition, current radiation-enhancing methodologies such as the combination of radiation with pharmacological therapies can have adverse effects on healthy tissues. As a result, there is an unmet need to develop and optimize a therapy that would effectively sensitize cancer cells to radiation and avoid systemic effects on normal tissues, while allowing the use of a relatively low radiation dose. Recent studies have demonstrated the use of ultrasound-stimulated microbubbles as a new biophysical therapy to enhance radiation effects. The ultrasound can be focused so that enhancement is specifically confined to tumor tissue. Here, the authors aimed to better characterize cancer cell responses to this novel type of radiation-enhancement therapy at the molecular level.

**Results**

Using immunohistochemical analyses, the authors evaluated the levels of a range of molecular markers associated with cellular processes that are potentially affected by combined ultrasound-based radiation-enhancing therapy. Markers associated with apoptotic signaling, proliferation, vasculature, cell-membrane damage, hypoxia and DNA damage were examined in xenograft models of prostate cancer. Apoptotic cell death was more evident in the combined treatments when compared with the single treatments, and cellular proliferation was significantly lower in the combined treatment with 8 Gy of radiation. Additionally, levels of vascular damage and leakage were increased and were accompanied by increased ceramide signaling, hypoxia and DNA damage in response to combined treatment.

**Implications and future directions**

This study characterizes the effects of ultrasound-stimulated microbubble therapy on cancer cells, illustrating the involvement of key cellular signaling pathways in the responses to treatment. The effects might be explained by the local biological effects of the stimulated microbubbles, which can include mechanical stresses and the production of reactive oxygen species, leading to endothelial cell damage, vascular disruption and collapse, and secondary tumor cell death. Importantly, this ultrasound-based enhancement therapy can be targeted to tumor cells only, making it selective. As well as enhancing radiotherapy, this could also enable the use of lower radiation doses to achieve an effect equivalent to current strategies. Future directions include evaluating the effects of multiple treatments and optimizing these in orthotopic tumors, in addition to scaling up treatments for larger tumors.

These recent advances have provided proof-of-principle evidence that ultrasound-stimulated microbubbles have a wide range of oncologic applications. However, investigations have not fully explored the use of microbubbles as biophysical tumor radiation-enhancing and -disrupting agents. In this study, we investigate in detail the use of ultrasound-driven microbubbles as a single therapeutic agent and as a combined treatment with radiation in a prostate cancer xenograft model. Characterization of tumor response was assessed by using clonogenic assays, histopathology, *in situ* end labeling (ISEL), terminal deoxynucleotidyl transferase dUTP nick-end labeling (TUNEL) and other immunohistochemistry assays. Specifically, detailed immunohistochemistry analysis combined with quantitative microscopy was used to detect changes in a number of cellular markers that are modulated by the therapy and can reflect the status of tumor vascularization, hypoxia and cell proliferation (Kamat et al., 2007; Jokilehto et al., 2006; Zlobec et al., 2009).

Results indicate that microbubble-stimulated radiation affected tumor vascularization and Ki-67 activity greater than radiation alone or ultrasound-stimulated microbubble treatment alone. The combined therapy resulted in the greatest destruction of tumor vasculature concomitant with the greatest detected extent of tumor cell death. The resultant tumor core exhibited hypoxia but, paradoxically, with an enhancement of radiation-induced cell death as assessed by immunohistochemistry and clonogenic cell survival assays.

## RESULTS

### Treatment effects on signaling, vasculature, oxygenation and DNA damage

**Fig. 1.**
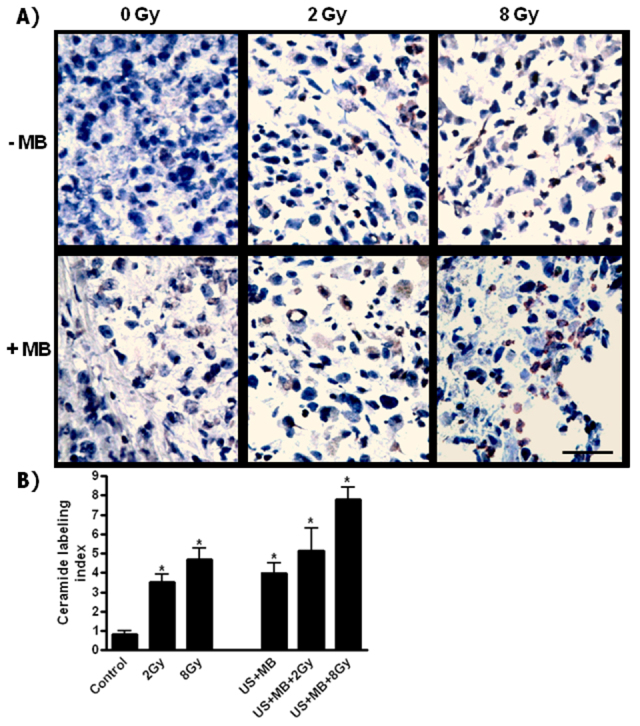
**Ceramide labeling of tumor sections.** (A) Brown-red labeling of ceramide increased in intensity and distribution with the combined treatments than the single treatments. (B) Labeling analyses using ImageJ indicated a significant difference (*) when comparing the labeling of the different treatment groups to the control (*P*=0011 with either 2 Gy alone or combined, *P*=0005 with either US+MB or 8 Gy alone; *P*=0002 with US+MB+8 Gy. A Mann-Whitney test was used to calculate the *P*-values. Scale bar: 50 μm.

Ceramide staining was investigated because previous research has indicated its role in signaling changes (Al-Mahrouki et al., 2012; Czarnota et al., 2012; Kim et al., 2013). Findings (Fig. 1) indicated increases in ceramide with microbubble exposure and with radiation. Effects were greatest in the treatment with ultrasound-stimulated microbubbles and 8 Gy radiation exposure (*P*<0.05).

**Fig. 2.**
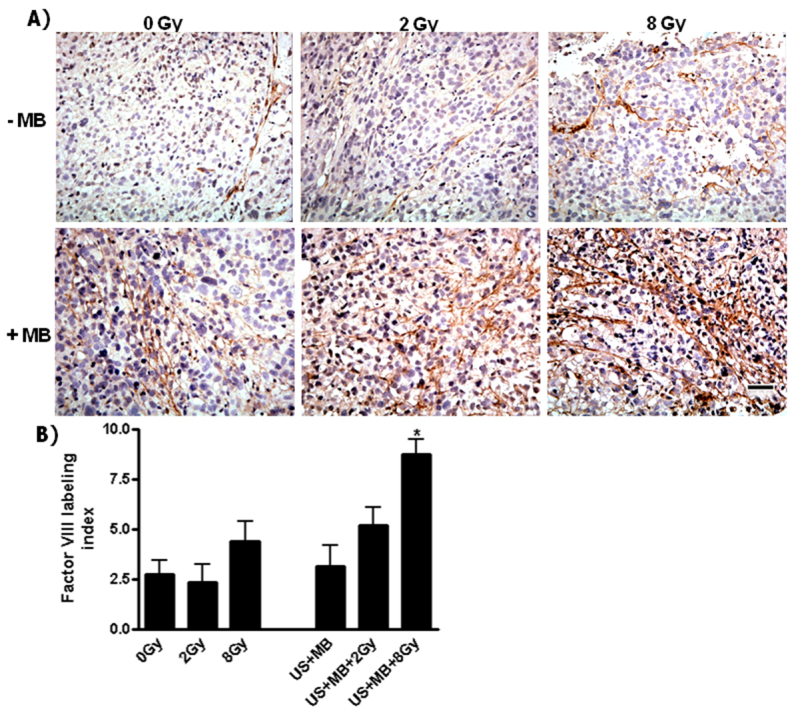
**Vessel integrity was detected using immunohistochemical labeling of factor VIII in PC3 xenograft sections.** (A) Micrographs of sections from tumors not treated with microbubbles (–MB; upper panels) and from tumors treated with microbubbles (+MB; lower panels). (B) Blood-vessel leakage became more evident and significant when the ultrasound-activated microbubbles were combined with a radiation dose of 8 Gy. Statistical analyses indicated a *P*<0.029 (*). Scale bar: 50 μm.

Tumor vascular damage associated with treatments was assessed by immunolabeling of clotting factor VIII to evaluate the extent of disruption and the resulting blood leakage (Fig. 2). Increased vascular damage was observed, which was associated with an increased vascular leakiness, and this damage was predominantly associated with the combined treatment of ultrasound-stimulated microbubbles and 8 Gy (*P*<0.029).

**Fig. 3.**
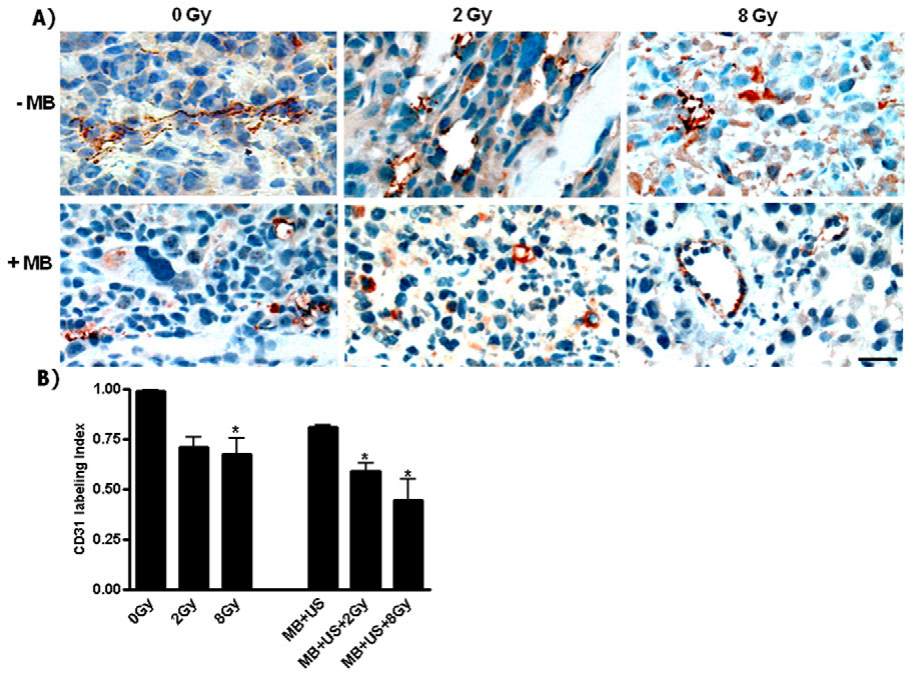
**Angiogenesis assessment with immunohistochemistry labeling of CD31.** (A) Micrographs of tumor sections, illustrating labeling of blood-vessel endothelial cells (brown-red) treated with different conditions. (B) Decreased vascularization was observed with the treatments with 8 Gy (*P<0.043), with MB+US+2 Gy (*P<0.032) and with MB+US+8 Gy (*P<0.01). Scale bar: 25 μm.

**Fig. 4.**
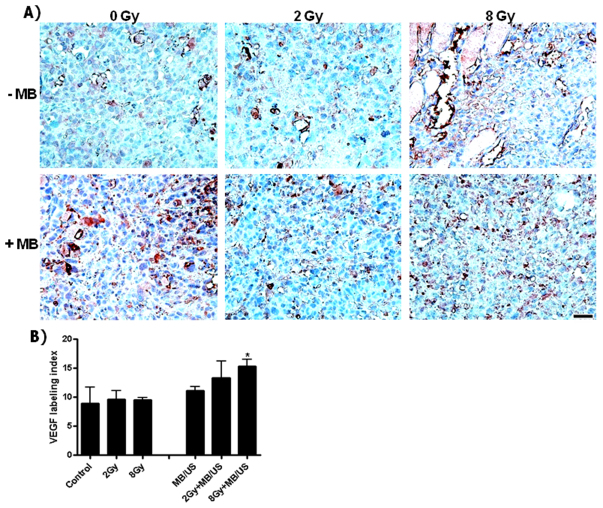
**Assessment of angiogenesis signaling using immunohistochemistry labeling of VEGF.** (A) Micrographs of tumor sections, illustrating labeling of blood-vessel endothelial cells (brown-red) that were treated with different conditions. (B) A significant signaling increase was observed with combined (MB+US+8 Gy) treatments (*P<0.032). Scale bar: 50 μm.

Changes in vascular index were investigated using CD31 immunohistochemistry, a cell-surface receptor expressed on the membrane of endothelial cells considered as a marker to measure angiogenesis. Vascular labeling was significantly decreased when either 8 Gy (*P*<0.043), or ultrasound-stimulated microbubble and 2 Gy (*P*<0.032), or when the combined treatment with 8 Gy (*P*<0.01) were used (Fig. 3), as assessed using the *t*-test. In order to investigate treatment effects on angiogenesis signaling, VEGF was assessed using immunolabeling. A significant signaling increase was observed with the combined treatment with 8 Gy (*P*<0.032) (Fig. 4 and supplementary material Fig. S1).

**Fig. 5.**
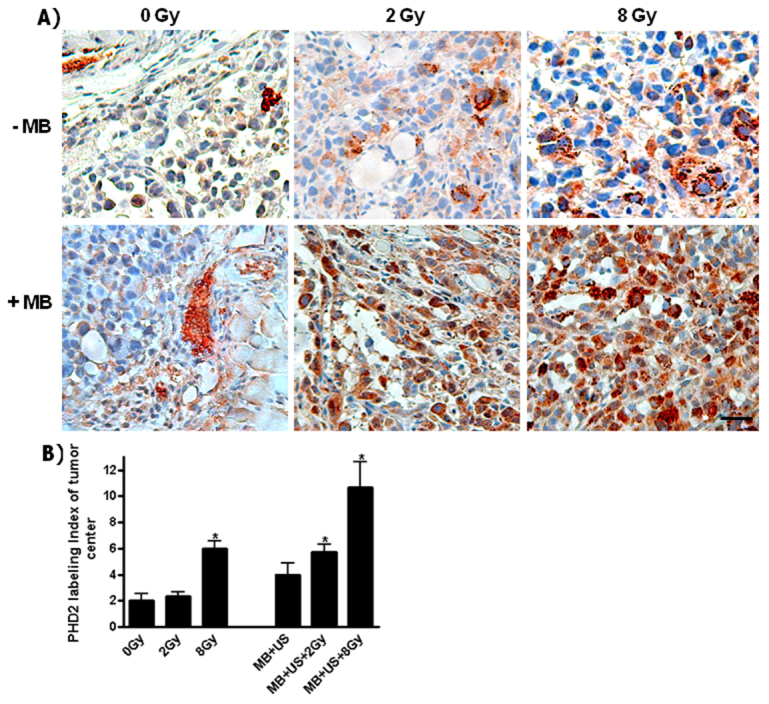
**Hypoxia staining using PHD2 immunohistochemistry of tumor sections.** (A) Labeled sections illustrated an increased staining with the combined treatments. (B) Statistical analyses revealed a significant change (*) when comparing the controls to 8 Gy (*P*<0.05), to MB+US+2 Gy (*P*<0.008) or to MB+US+8 Gy (*P*<0.012). Scale bar: 25 μm.

Because these vascular treatments can affect oxygenation of tissue, hypoxia was evaluated by staining for PHD2, an oxygen-sensing molecule that modulates hypoxia-inducible factor (HIF) response under low oxygen levels. Labeling of PHD2 was observed using immunohistochemistry in tumor cells and endothelial cells with exposure to different treatments (Fig. 5). An increase in the level of PHD2 in the center of the treated tumors was observed when treating with the higher radiation dose of 8 Gy (*P*<0.05), or with the ultrasound-stimulated treatments combined with radiation [microbubble (MB)+ultrasound-stimulated (US)+2 Gy (*P*<0.008) or MB+US+8 Gy (*P*<0.012)].

**Fig. 6.**
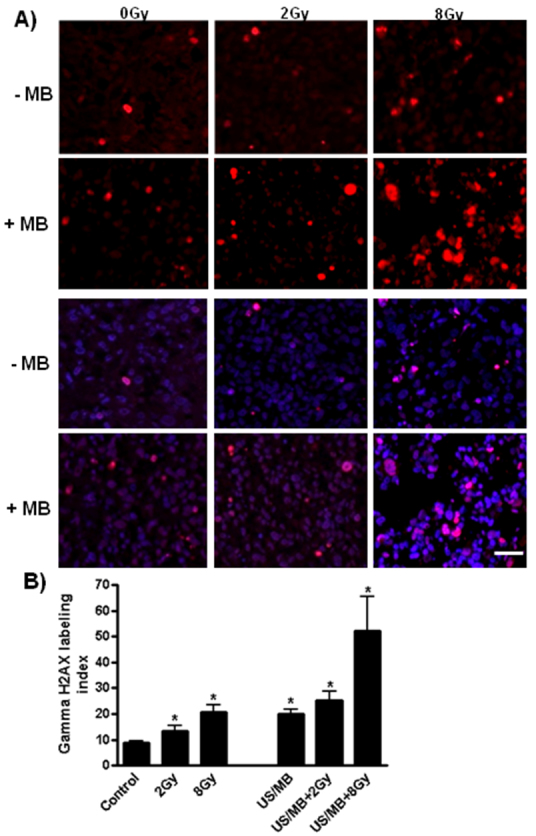
**Staining for DNA damage.** (A) Immunostaining of γH2AX (red fluorescence; upper two panels) and overlay with DAPI as a counter stain (blue fluorescence; lower two panels). (B) Increased labeling was observed with all the treatments, with *P<0.029 (MB+US and MB+US+2 Gy) and *P<0.014 (2 Gy, 8 Gy and MB+US+8 Gy). Scale bar: 30 μm.

The effects of ionizing radiation were evaluated by staining with antibodies against γH2AX (Fig. 6), which is a histone subtype associated with DNA damage. Immunolabeling of γH2AX revealed significantly elevated levels of γH2AX production under the different treatments (*P*<0.029, ultrasound-stimulated microbubbles alone and combined with 2 Gy, and *P*<0.014, for treatments with 2 Gy, 8 Gy and ultrasound-stimulated microbubbles combined with 8 Gy). A significant increase in γH2AX was also observed when comparing single 2 Gy treatments to the combined treatment of ultrasound-stimulated microbubbles (*P*<0.029) or when comparing 8 Gy to the combined therapy involving 8 Gy (*P*<0.014) with the combined treatments demonstrating more staining. This was further supported by one-way ANOVA testing with a *P*<0.002.

### Cell death, survival and proliferation

**Fig. 7.**
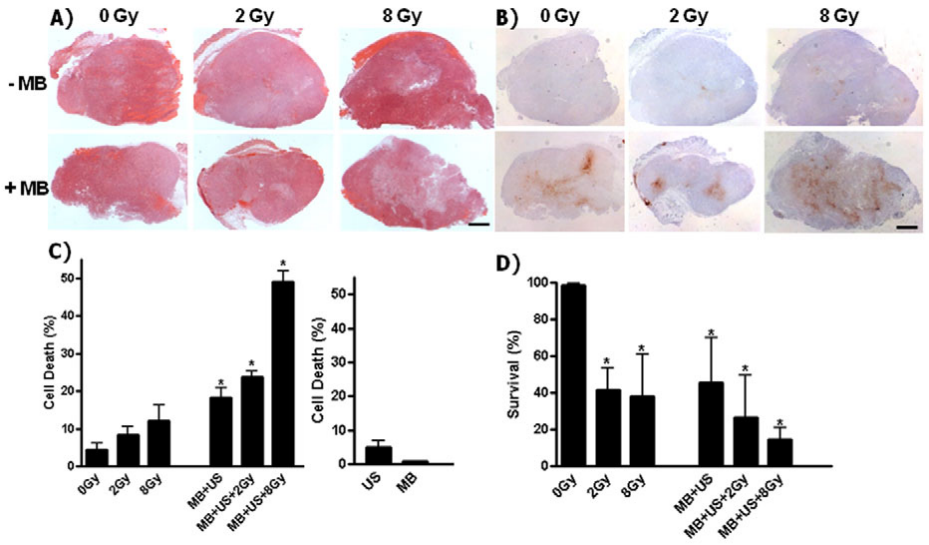
**Histopathology, *in situ* end labeling (ISEL) and clonogenic assays of a PC3 xenograft tumor.** (A) H&E staining of whole tumor sections treated with 0, 2 and 8 Gy or with a combination of radiation and ultrasound-stimulated microbubbles (–MB indicates no exposure to ultrasound-stimulated microbubbles; +MB indicates treatment with ultrasound-stimulated microbubbles). (B) Sections adjacent to those in A were labeled with ISEL to illustrate areas of cell death. Scale bars: 1 mm. (C) Quantified analyses of ISEL images, indicating an increased level of cell death with the combined treatments. A Mann-Whitney test was used to calculate the *P*-values and * symbols indicate where *P*-values are less than 0.05. (D) Clonogenic assay results illustrated a significant decrease in cellular survival of treated tumor cells when compared to the untreated samples. This was greatest in the combined treatments. A Mann-Whitney test was used to calculate the *P*-values and * symbols indicate where *P*-values are less than 0.05.

Results revealed increases in cell death with enhancement apparent when ultrasound-stimulated microbubble treatments (US+MB) were combined with either 2 Gy or 8 Gy radiation doses (US+MB+2 Gy, US+MB+8 Gy). Tumor disruption and cell death was apparent in hematoxylin and eosin (H&E)-stained sections as a white blanched central area (Fig. 7A) with corresponding ISEL staining (Fig. 7B). Cell death approached 23.8±1.5% and 49.2±2.9% when ultrasound-stimulated microbubble treatment was combined with 2 Gy and 8 Gy radiation doses, respectively. On their own, radiation treatments caused minimal increases in cell death (Fig. 7C). Analyses indicated that treatment-induced cell death levels were significantly different when comparing the control with bubble-alone treatment (*P*<0.05) or when comparing the combined treatments with 2 Gy (*P*<0.029), or with 8 Gy (*P*<0.012) treatments alone. In contrast, single treatments of 2 Gy or 8 Gy did not reveal significant differences from the control. Control treatments with ultrasound in the absence of microbubbles, or microbubbles administered in the absence of ultrasound stimulation, caused no appreciable effect.

Clonogenic survival results for a single treatment (radiation alone, ultrasound-stimulated microbubbles alone, or the combination) are given in Fig. 7D). Results demonstrated that the combination of the ultrasound-stimulated microbubbles and radiation doses had less survival than either of the single modalities used for treatment alone. For the single treatments with 2 Gy, 8 Gy or ultrasound-stimulated microbubbles, we observed cell survival ranging between 45.5±24.8% to 38.2±22.8%. Cell survival decreased with the combined treatments to 26.8±22.7% and 14.4±6.9% for the treatments with 2 Gy and 8 Gy, respectively. Data were significant when compared to the control (*P*<0.05). Statistical analyses using the Mann-Whitney test showed significant *P*-values when compared to the control groups. A significant difference was found between the control and 2 Gy (*P*<0.008), 8 Gy (*P*<0.018) and US+MB (*P*<0.048) conditions. Differences were also present between the control and combined treatment of US+MB+2 Gy with *P*<0.018, or the treatment with US+MB+8 Gy with *P*<0.008. One-way ANOVA was also used, demonstrating a significant change with *P*<0.0095 (Fig. 7D).

**Fig. 8.**
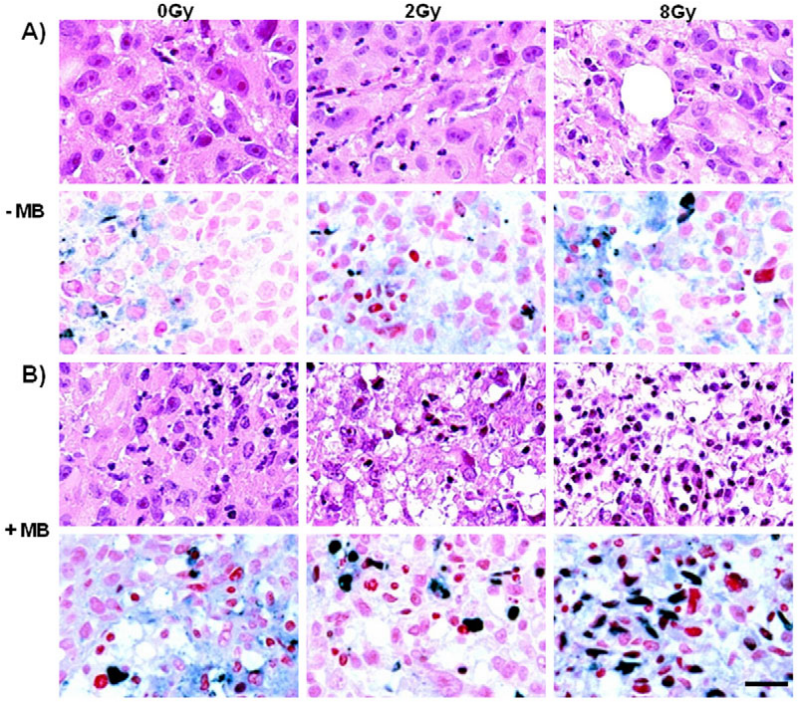
**Histopathology and terminal deoxynucleotidyl transferase dUTP nick-end labeling (TUNEL) assays.** The upper panels in A and B represent micro-photographs from sections stained with H&E. These demonstrate very noticeable morphological changes with the combined conditions of ultrasound-stimulated microbubbles and radiation. Cell death retraction artefact is noticeable in addition to condensed appearing pyknotic nuclei. Images in the lower panels in A and B are from sections adjacent to those in the upper panels and were labeled with the TUNEL assay. These illustrate increased nuclear condensation (red) and DNA fragmentation (dark blue) with combined treatment, which was quite obvious in areas of cell death, which were indicated by cellular morphological changes. Scale bar: 25 μm.

Higher-magnification inspection of H&E- and TUNEL-stained tumor sections revealed that the combination of ultrasound-stimulated microbubble and radiation treatments induced cellular apoptosis. Prominent retraction artefact or areas of a cellular destruction were evident with combination therapy exposure (Fig. 8). Cellular damage was mostly confined to the center of the tumors or within defined tumor regions that seemed to be associated with vasculature. Histopathology indicated both mixed apoptotic and necrotic morphologies (Fig. 8) with cells exhibiting ruptured membranes (necrotic cells) as well as condensed and fragmented nuclear material (apoptotic cells). Results were generally consistent with colony assay data and indicated less cell survival with the combined treatments than with any of the single treatments.

In order to investigate the response of a number of essential biological processes that are necessary for the maintenance of tumor cells, factors such as cellular proliferation, vascular leakage, angiogenesis, hypoxia and levels of DNA damage analysis were assessed using immunohistochemistry.

**Fig. 9.**
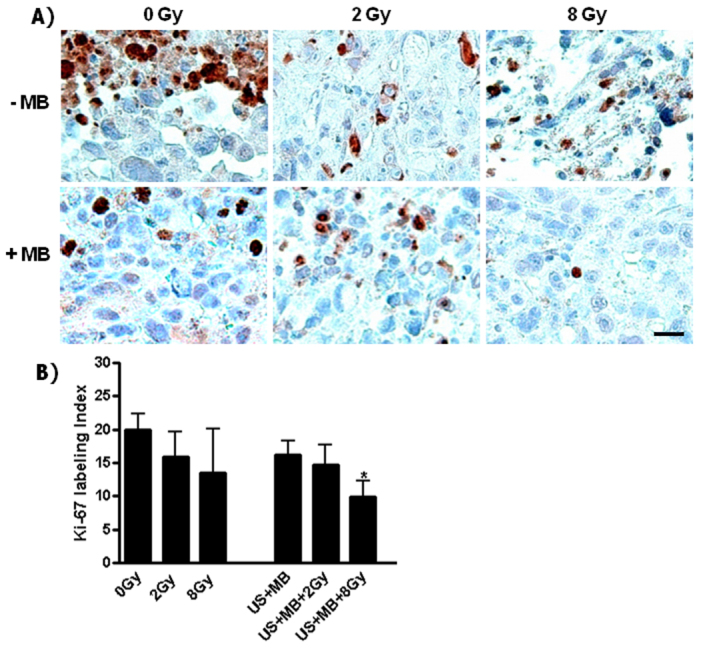
**Detection of cellular proliferation using Ki67 as a marker.** (A) An increased number of labeled nuclei were observed in the controls than in the treated samples. This indicated a decreased proliferation specifically with the combined treatment of ultrasound-stimulated microbubbles and radiation, where a significant difference was found *P*<0.024 (see B, *). (B) The number of positively stained nuclei was counted in whole sections and the number of the labeled cells/mm^2^ was calculated and plotted. A Mann-Whitney test was used to calculate the *P*-values. Scale bar: 25 μm.

Investigation of tumor cell proliferation using anti-Ki67 antibodies (Ki67 is a proliferation marker) assessed nuclear staining in tissues exposed to the different treatments. There was less nuclear staining for the combined treatments with either 2 Gy or 8 Gy, when compared to the control or to the other treatments (Fig. 9). Statistical analysis using the Mann-Whitney test indicated that the combined treatments were significantly different from those of the controls. The 2 Gy treatment combined with ultrasound-stimulated microbubbles (US+MB+2 Gy; 15±3 Ki67+ cells/mm^2^) compared to ultrasound-stimulated microbubble only (23±2 Ki67+ cells/mm^2^) was statistically significantly different (*P*<0.033). Comparing the combined 8 Gy treatment (10±2 Ki67+ cells/mm^2^) to the untreated control (20±2 Ki67+ cells/mm^2^) resulted in *P*<0.024; comparison to the ultrasound-stimulated microbubble treatment (US+MB+8 Gy) resulted in *P*<0.004. The single treatments with radiation or ultrasound-stimulated microbubbles did not reveal significant differences from the controls (Fig. 9B). Because ultrasound stimulation alone at an acoustic pressure of 570 kPa caused no appreciable effect either in *in vitro* or *in vivo* experiments that were carried out in our laboratory, it is unlikely that the observed effects in the combined treatments could result if bubbles were excluded. With *in vitro* experiments, exposure to ultrasound and the pressures used result in no observable bioeffect in terms of clonogenic assays. Xenograft tumors exposed to ultrasound alone also have no observable changes in terms of the immunohistochemistry markers studied here. It is possible that, at higher ultrasound pressures that cause cavitation in the absence of microbubbles, a similar effect could be observed.

## DISCUSSION

The study here characterized the effects of using ultrasound-driven microbubbles in combination with radiation. This treatment is believed to cause a mechanical disruption of tumor vasculature leading to enhanced cellular damage when combined with radiation (Al-Mahrouki et al., 2012; Czarnota et al., 2012; Tran et al., 2012; Kim et al., 2013). Previous research has characterized this effect macroscopically in a limited manner using *in vitro* models, and in regards to changes in gene expression and cellular morphological appearance (Al-Mahrouki et al., 2012; Nofiele et al., 2013). Furthermore, combining ultrasound-activated microbubbles and other therapeutic approaches such as chemotherapy has also proven to be significantly effective in therapy sensitization (Goertz et al., 2012). That work, despite the relatively high pressure of 1.65 MPa that was used as compared to 570 kPa which was used in this study, also has results that are supportive of ultrasound-stimulated microbubbles inducing vascular disruption, which leads to enhanced tumor responses to anti-cancer therapies similar to those effects previously reported (Czarnota et al., 2012). Interestingly, the use of a fractionated low radiation dose that is below the clinical effective dose was shown to cause greater tumor shrinkage when combined with bubble treatment (Czarnota et al., 2012). There, the effects of a non-curative dose of radiation [biological effective dose (BED_10_)=29 Gy] combined with microbubble treatments was more effective than a curative dose of radiation (BED_10_=58 Gy).

This is specifically important for the translational approach, where healthy cells would be spared the damaging effect of a high radiation dose. However, potential pathways and mechanisms involved in such therapies are yet to be elucidated and are the focus of the fine-level immunohistochemistry work done in this study.

Here we have undertaken an extensive analysis of treatments in which ultrasound-stimulated microbubble treatments of tumors are combined with radiation, in order to assess treatment effects on cells and tumor tissue. Specifically, we used immunohistochemical methods to probe changes in biological markers associated with ultrasound stimulation of microbubbles and their combination with radiation. Results here, in general, have indicated elevated levels of tumor cell death when tumors were exposed to ultrasound-stimulated microbubbles. This has been demonstrated previously for bladder and prostate tumor lines (Tran et al., 2012; Czarnota et al., 2012). In the study here we carefully characterized the effects of these treatments at a cellular level using immunolabeling, focusing on the most extensively studied tumor type (prostate cancer). Results indicate changes in levels of cellular proliferation, changes in the extent of vasculature, changes in oxygenation of tissue and altered levels of DNA damage with the use of ultrasound-stimulated microbubbles, when used in combination with radiation.

Specifically, in the mechanism posited for the combination of ultrasound-stimulated microbubbles and radiation treatments, mechanical stimulation of endothelial cells when combined with radiation causes vascular disruption in excess of that capable of being induced by a single modality treatment. It is believed that this then leads to secondary death of tumor cells. The amount of death seems to be in excess of that elicited by comparable or even higher doses of radiation, which can also have vascular effects (Paris et al., 2001; Garcia-Barros et al., 2003). Effects visualized in this study were consistent with previously published effects on specimens analyzed with H&E staining or samples assessed for apoptosis. The combined treatments involving ultrasound-stimulated microbubbles and radiation indicated more cell death, as expected.

In regards to vascular effects, in this study we observed a significant decrease in CD31 immunolabeling, specifically with the combined treatments, and a concurrent significant increase in vessel leakage when evaluated using immunolabeled clotting factor VIII. In the treatments that caused more damage, treatment effects were also associated with increased levels of VEGF. Many approaches have investigated the disruption of angiogenesis as a promising treatment strategy, targeting VEGF or integrins as a potentially effective anticancer therapy. Nevertheless, the use of anti-VEGF drugs (Ebos et al., 2009; Pàez-Ribes et al., 2009) or anti-integrins (Reynolds et al., 2009) can paradoxically demonstrate a faster spread of tumors and an adverse response such as the initiation of angiogenesis. A study by Cervi et al. (Cervi et al., 2007) also reported that VEGF overexpression can suppress biological processes such as leukemogenesis. In addition, pathways involved in neovascularization have been reported to involve other microenvironment signals such as physical interactions between cells and the extracellular matrix (Matthews et al., 2006; Huang and Ingber, 1999) that can influence the role of CD31 in vascular growth (Mammoto et al., 2009). This suggests that the effects on VEGF here are related to the effects on vasculature that the ultrasound-stimulated microbubble treatments might have.

Treatments with ultrasound-stimulated microbubbles alone in tissues, but using high pressure ultrasound with peak negative pressures such as 2.3 MPa, can also lead to vascular effects. These are manifest primarily as significant hemorrhage in conjunction with vascular disruption (Miller et al., 2011). In the work here, we used a lower pressure of 570 kPa, which caused endothelial cell death and vascular collapse as before (Czarnota et al., 2012). Ceramide production was investigated here, as a marker for cell stress. Results indicated increases with the combined treatments. Levels of microbubble-induced damage were reflected in increased ceramide production with the combined treatments, consistent with previous observations (Al-Mahrouki et al., 2012; Czarnota et al., 2012; Nofiele et al., 2013). The final circulating number of bubbles in this study was about 1.5×10^6^ (~0.3% of mouse blood volume), which is equivalent to 84 μl/kg body weight. For clinical imaging, the concentration used is 21.7 μl/kg body weight. The effect appears to be related to the total number of bubbles burst (Kim et al., 2013). Hence, although we have used a higher concentration than that used for clinical imaging, it is possible to elicit similar treatment effects. We also note that the insonification here have only used 750 ms over 5 minutes, providing ample time to increase exposure.

We also investigated the effects that the treatments in this study can have on hypoxia, given its relationship to changes in VEGF levels (Cébe-Suarez et al., 2006) and that, when oxygen levels fall below 5%, hypoxia can also induce new vessel formation (Mazzone et al., 2009). We carried out staining specifically for PHD2, which is an oxygen-sensing molecule. Specifically, this protein catalyzes the post-translational formation of hydroxyl-proline and leads to the degradation of hypoxia-inducible factor (HIF1α) (Mazzone et al., 2009). As expected, the combined treatments in particular caused an induction in this molecule, consistent with previous evidence demonstrating vascular destruction with such treatments (Czarnota et al., 2012). Low oxygen levels induce HIF, which, in turn, induces angiogenesis. When HIF levels increase, PHD2 activities increase to degrade HIF to achieve homeostasis. Specifically, our observations of the immunolabeling of PHD2 in treated tumors showed its expression in both endothelial cells and tumor cells. The treatments being administered are noted to have effects on the vasculature. The greatest staining with PHD2 was noted in the combined treatments, which were also the treatments which had the greatest disruptive effect on the vasculature, as detected through CD31 and VEGF staining. In agreement with these observations, a recent investigation that used photoacoustic imaging by Briggs et al. (Briggs et al., 2013) has illustrated a significant decrease in oxygen saturation levels when treating prostate tumors with a combined therapy of microbubbles and 8 Gy, and as illustrated in supplementary material Fig. S2.

Cellular proliferation is also an important aspect in the evaluation of the effect of treatments on tumor regression. Accordingly, we investigated Ki67 as a proliferation marker. This marker is a nuclear protein that is expressed in all phases of active cells, but not in resting cells (Kee et al., 2002; Gravdal et al., 2009). Results revealed a significant decrease in cellular proliferation with the combined treatments, which was consistent with TUNEL and ISEL results, showing the greatest extents of cell death for these treatments. The Ki67 results were also consistent with colony assays for viability in which the greatest inhibition of Ki67 activity was in samples which had the lowest survival. In general, Ki67 activity is indicative of tumor resistance to treatment such as in the case of the castration-resistant prostate cancers (Gravdal et al., 2009).

In addition, because ISEL and TUNEL results indicated increased levels of cell death through the labeling of the fragmented DNA, we corroborated these results by investigating the production of γH2AX, which is a biomarker that is involved in DNA-break recognition and repair (Lukas et al., 2011). Its levels were significantly elevated with all the treatments when compared to the control, and also when comparing the single treatments of 2 Gy or 8 Gy to the ultrasound-stimulated microbubble treatments combined with radiation, where γH2AX levels were higher with the combined condition. The ultrasound and microbubble treatments given, followed by 8 Gy, indicated a significant increase in γH2AX labeling, indicative that tumor cells might be potentially sensitized by the vascular insult caused by ultrasound-stimulated microbubble enhancement of radiation effect. This could be explained by the changes in gene expression that can be induced by such microbubble stimulation (Al-Mahrouki et al., 2012) or, alternatively, by the fact that microbubble collapse can lead to the production of free radicals, which can cause cellular damage.

**Fig. 10.**
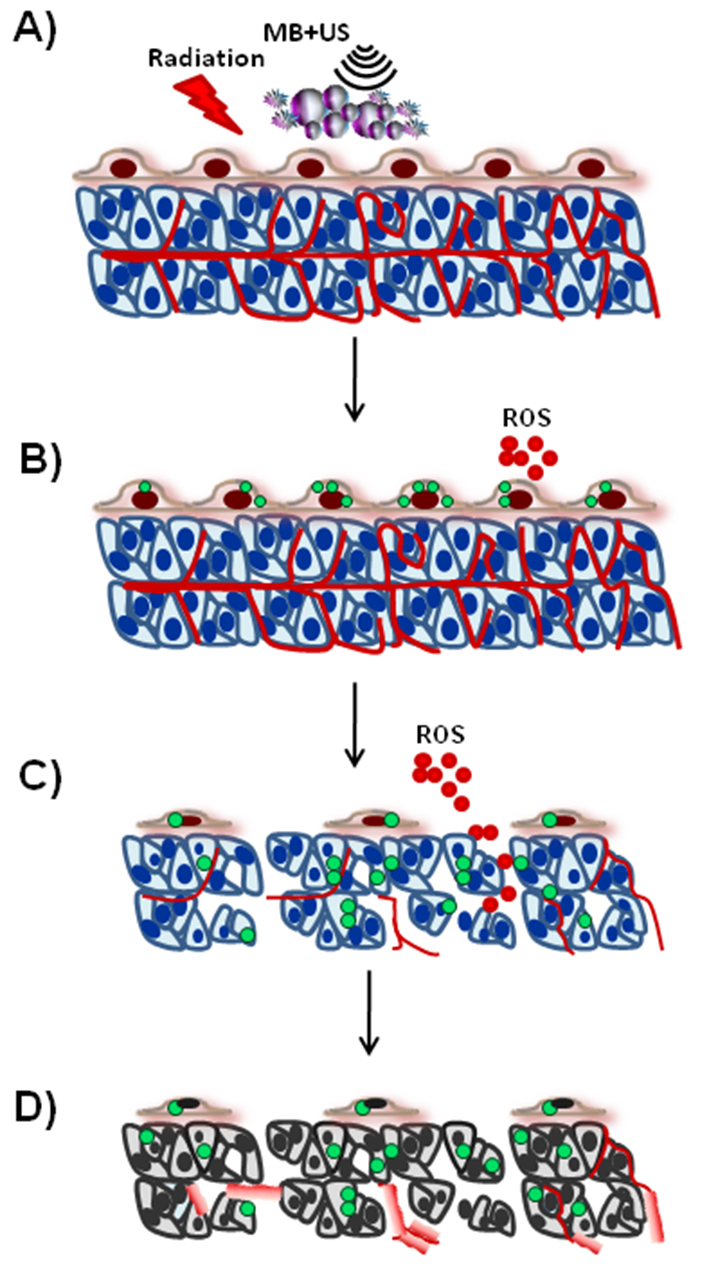
**Summary model of the response to the combined ultrasound-stimulated microbubble radiation-enhancing treatments.** Combining ultrasound-stimulated microbubble therapy with radiation (A) results in the induction of ceramide production (B; green circles) and the potential release of reactive oxygen species (B; ROS, red circles). This microbubble-stimulated damage eventually leads to disruption of vasculature (C) with enhanced secondary tumor cell death. Structural alterations also result in vascular leakage (D) and DNA damage secondary to apoptotic and ischemic tumor cell death (gray cells) (D).

In summary, the combined treatments of ultrasound-stimulated microbubble treatment with radiation, known to result in enhanced response of tumor cells to radiation, caused changes in tumor vasculature and the cellular microenvironment as demonstrated here, through immunohistochemical staining analyses. Changes occurred in immunolabeling markers linked to cell death. Changes were also apparent in Ki67, which is linked to cellular proliferation. Vascular changes were also shown, as detected through CD31 labeling and factor VIII staining. Changes were also apparent in γH2AX in tumor cells, showing an enhancement of effect induced by ultrasound-microbubble stimulation of endothelial cells leading to increased vascular destruction. A schematic of the gross changes related to these observations is provided in Fig. 10.

In summary, immunohistochemistry carried out here to probe molecular level changes in key response elements in addition to tumor cell morphology demonstrated changes consistent with vascular disruption caused by the combined ultrasound-stimulated microbubble and radiation treatments. Effects on vascular integrity were present, linked to decreases in vascular index and altered VEGF expression. Treatments in which ultrasound-stimulated microbubbles were used in combination with radiation indicated greater levels of hypoxia but also greater levels of ceramide production, cell death and DNA damage than caused by single treatments. Other research has demonstrated that multiple treatments of ultrasound-stimulated microbubbles result in superior animal survival when used for tumor cure, pointing towards mechanisms of vascular disruption and secondary ischemic cell death rather than hypoxia as a major treatment effect (Czarnota et al., 2012). The treatments here also had effects on cell proliferation and clonogenic cell survival, demonstrating greater effects when used together rather than as single modalities alone. These findings were consistent with increases in cell death with the combined treatments, as assessed histologically.

Treatments were found to induce hypoxia as before. It is possible that increased hypoxia in malignant prostate tumors could result in lowering the levels of survival in clonogenic assays and make cells more aggressive (Butterworth et al., 2008). However, we also cannot rule out the effect of cell trauma resulting from cellular dissociation after treatment making such cells less aggressive. Here we have taken the approach of causing hypoxias at levels that are cytotoxic (anoxia). By destroying blood vessels, blood flow is shut off to tumor cells, resulting in apoptosis-necrosis and decreased tumor survival. Experiments *in vivo* with this methodology have resulted in superior animal survival, pointing to therapeutic benefit (Czarnota et al., 2012). Other researchers have investigated normalizing blood vessels to reverse hypoxia as a means of increasing sensitization of tumor to radiation, a promising approach but which still needs further optimization. Here we report increased levels of hypoxia that exceed thresholds of survival and lead to cell death.

The research here was carried out using xenograft models of prostate cancer. It is appreciated that orthotopic tumor models might more accurately reflect vasculature in human tumors. Differences in ultrasound parameters might be required to account for any differential vascular structure and properties.

In conclusion, the results provide a basis for understanding the morphological and mechanistic effects of these new ultrasound-microbubble-based treatments, which significantly enhance cell death caused by radiation.

## MATERIALS AND METHODS

### Cell culture

Prostate cancer cells (PC3, American Type Culture Collection, Manassas, VA, USA) were cultured in RPMI-1640 (Wisent Inc., St Bruno, Canada) culture media, which included 10% fetal bovine serum (FBS; Thermo Fisher Scientific, Waltham, USA) and 100 U/ml of penicillin/streptomycin (Invitrogen, Carlsbad, USA). Cells were grown and maintained under humidity at 37°C, 5% CO_2_. Confluent cells were harvested using 0.05% Trypsin-EDTA (Invitrogen, Carlsbad, USA). Cells were collected by centrifugation at 4°C for 10 minutes (200 ***g***) and were resuspended in phosphate buffer saline (PBS) in preparation for animal injection.

### Treatments

Five- to six-week-old CB-17 severe combined immunodeficiency (SCID) male mice (Charles River Laboratories International, Wilmington, MA, USA) had xenograft tumors induced by injecting 1×10^6^ PC3 cells suspended in 50 μl of PBS subcutaneously in the upper hind legs of the animals. Tumors were allowed to develop to a diameter of 7–10 mm within approximately 1 month from the initial time of induction.

Animals were anesthetized prior to treatment by an intraperitoneal injection of a mixture of Ketamine (100 mg/kg body weight), Xylazine (5 mg/kg body weight) and Acepromazie (1 mg/kg body weight) (Sigma, Burlington, ON, Canada). The treatments included: radiation alone (0 Gy, 2 Gy, 8 Gy), ultrasound-stimulated microbubbles (0.3% v/v) alone and a combination with the ultrasound-microbubble treatments followed immediately by radiation. Eight animals were used per condition. Definity microbubbles (Lantheus Medical Imaging, N. Billerica, MA, USA) were activated by shaking for 45 seconds at 3000 rpm using a Lantheus Vial-shaker device. The therapy set up consisted of a wave-form generator (AWG520, Tektronix), an amplifier (RPR4000, Ritec), an acquisition system (Acquiris CC103) and an ultrasound transducer with 500 kHz center frequency and 28.6 mm aperture diameter. It was focused at 85 mm, and the −6 dB width of the focal zone was 31 mm. It was purchased from Valpey Fisher Inc. (Hopkinton, MA; Cat#IL0509HP). The ultrasound beam and its field for this transducer are shown in a map in the supplementary section (supplementary material Fig. S3).

As was previously described (Czarnota et al., 2012; Tran et al., 2012), the tumor on the hind leg was immersed into a 37°C water bath and was positioned within the half-maximum peak of the acoustic signal from the transducer. Tumors were exposed to 16 cycles tone burst of 500 kHz frequency with 3 kHz pulse repetition frequency for 5 minutes, resulting in 750 ms of exposure for an overall duty cycle of 0.25%. The duty cycle was designed to permit tumor refill between insonification, which causes bubble bursting *in vivo*. The peak negative acoustic pressure was 570 kPa (mechanical index of 0.8). For radiation treatments, mice were shielded with a lead sheet, except for the tumor region, which was exposed to radiation through a confined circular aperture. A CP-160 cabinet X-radiator system (Faxitron X-ray Corporation, IL, USA) was used to deliver 0, 2 or 8 Gy at a rate of 200 cGy/minute. Animals were sacrificed 24 hours after treatment and tumors were harvested and fixed in 1% paraformaldehyde or embedded in optimal cutting temperature (OCT) media, then flash frozen in liquid nitrogen and stored at −80°C for future analyses.

Procedures involving experimental animals were performed in compliance with local animal welfare laws, guidelines and policies.

### Clonogenic assays

Excised tumor portions were mechanically and chemically dissociated as previously described elsewhere (Dow et al., 1982). Tumor cells were passed through a series of needles (18-, 20- 22-gauge) and were then trypsinized with 0.25% Trypsin at 37°C for 10–15 minutes. Media (RPMI-1640) supplemented with 10% FBS was then added and cells were washed twice by resuspension and centrifugation at 450 ***g***. Cells were counted using a hemocytometer and 10^5^ cells were plated in triplicate and incubated at 37°C and 5% CO_2_ for 7 days to develop colonies. Colonies were then fixed and stained with 0.3% methylene blue/methanol for 20 minutes. The numbers of the counted colonies were compared and analysis by the Mann-Whitney test was used to determine the statistical significance.

### Histology and immunohistochemistry

Specimens were fixed in freshly prepared in 1% paraformaldehyde for up to 2 hours at room temperature then incubated at 4°C for 48 hours, after which fixative was replaced by 70% ethanol. Samples were then embedded in paraffin and 5-μm sections were placed on glass slides in preparation for staining. Histopathology was evaluated using hematoxylin and eosin (H&E) staining as well as *in situ* end labeling (ISEL) (Wijsman et al., 1993) and terminal dUTP nick-end labeling (TUNEL) staining using the *In Situ* Apoptosis Detection kit (R&D Systems, Minneapolis, USA), and was carried out according to the manufacturer’s instructions. Fragmented DNA was detected by labeled nucleotides (BrdU-labeled dNTPs), which were added to the 3′OH end by a terminal DNA transferase. Labeled tissue was incubated with anti-BrdU antibody, then with streptavidin-HRP, and the formed complex then labeled by TACS blue. The resulting dark blue nuclear staining was used to detect apoptotic nuclear chromatin. Nuclear Fast Red was used as a counter stain to stain all cells with normal cells pale pink, whereas apoptotic condensed cells stained red or purple. Areas of cell death within the sections of the tumors were measured using ImageJ (National Institutes of Health, Bethesda, Maryland, USA).

Immunolabeling was performed using a Histostain-plus kit (Invitrogen, Carlsbad, CA), following the manufacturer’s instructions. Tissue sections were deparaffinized in xylene and dehydrated in a graded ethanol series, and washed in PBS. In order to unmask the antigenic sites, tissues were treated with 10 mM sodium citrate buffer and incubated at 95–100°C for 20–40 minutes. Tissues were left to cool at room temperature for 20 minutes. Slides were then washed with PBS and the endogenous peroxidase was quenched by 3% hydrogen peroxide in methanol. In order to block the nonspecific background, 10% non-immune serum (goat) was used, which was followed by the incubation of the sections with the primary antibody for 1 hour at room temperature. A biotinylated secondary antibody was used, followed by incubation with horseradish peroxidase conjugated to streptavidin. This formed complex was then labeled by AEC (3-amino-9-ethylcarbazole substrate chromogen), creating an intense red color. Hematoxylin was used as a counter stain. All stained tissues were imaged using a LEICA DM LB light microscope and Leica IM1000 software. Primary antibodies (Abcam, Cambridge, MA, USA) used for the immunostaining included polyclonal to CD31 (mouse) used at 0.2 mg/ml, polyclonal to VEGF (human) at 0.5 mg/ml, polyclonal to PHD2/prolyl hydroxylase (human) at 1 mg/ml and polyclonal to Ki67 (human) at 1 mg/ml. All antibodies were used at a dilution ration of 1:20 except for the antibody Ki67 which was used at a 1:10 dilution. Factor VIII and γH2AX immunolabels were done by the Biomarker Imaging Research Laboratory at Sunnybrook Health Sciences Centre (Toronto, Canada).

In order to assess proliferation with Ki67, immunostained cells were counted throughout each tumor section and the total area of each section was then calculated to find the number of proliferating cells/mm^2^. A vascular index with CD31 staining was similarly determined. The results were then averaged and compared using a Mann-Whitney test or *t*-test to determine statistical significance.

For ceramide staining, tumor tissues embedded in OCT medium were snap frozen in liquid nitrogen then stored at −80C. Frozen 8-μm sections were then prepared and used for ceramide labeling after washing the sections with PBS at room temperature. Immunolabeling was then carried out as was previously described (Al-Mahrouki et al., 2012).

For the immunolabeling of PHD2, VEGF, factor VIII, CD31, ceramide and γH2AX, the quantification of the staining was done using either ImageJ (National Institutes of Health, Bethesda, Maryland, USA; Immuno-Ratio) or a tally counter. Data were collected from three to six mice per treatment condition, and three to five random regions of interest were imaged at 20× magnification, representing a tumor section, were analyzed; resulting data were then averaged. With the exception of Ki67 and ISEL analyses, data were collected from whole tumor sections.

## Supplementary Material

Supplementary Material

## References

[b1-0070363] Al-Mahrouki A. A. Karshafian R. Giles A. Czarnota G. J. (2012). Bioeffects of ultrasound-stimulated microbubbles on endothelial cells: gene expression changes associated with radiation enhancement in vitro. Ultrasound Med. Biol. 38, 1958–1969 22980406 10.1016/j.ultrasmedbio.2012.07.009

[b2-0070363] Badea R. Seicean A. Diaconu B. Stan-Iuga R. Sparchez Z. Tantau M. Socaciu M. (2009). Contrast-enhanced ultrasound of the pancreas – a method beyond its potential or a new diagnostic standard? J. Gastrointestin. Liver Dis. 18, 237–242 19565060

[b3-0070363] Bao S. Wu Q. McLendon R. E. Hao Y. Shi Q. Hjelmeland A. B. Dewhirst M. W. Bigner D. D. Rich J. N. (2006). Glioma stem cells promote radioresistance by preferential activation of the DNA damage response. Nature 444, 756–760 17051156 10.1038/nature05236

[b4-0070363] Bastianutto C. Mian A. Symes J. Mocanu J. Alajez N. Sleep G. Shi W. Keating A. Crump M. Gospodarowicz M. (2007). Local radiotherapy induces homing of hematopoietic stem cells to the irradiated bone marrow. Cancer Res. 67, 10112–10116 17974951 10.1158/0008-5472.CAN-07-2192

[b5-0070363] Becher O. J. Hambardzumyan D. Walker T. R. Helmy K. Nazarian J. Albrecht S. Hiner R. L. Gall S. Huse J. T. Jabado N. (2010). Preclinical evaluation of radiation and perifosine in a genetically and histologically accurate model of brainstem glioma. Cancer Res. 70, 2548–2557 20197468 10.1158/0008-5472.CAN-09-2503 PMC3831613

[b6-0070363] Bos P. D. Zhang X. H. Nadal C. Shu W. Gomis R. R. Nguyen D. X. Minn A. J. van de Vijver M. J. Gerald W. L. Foekens J. A. (2009). Genes that mediate breast cancer metastasis to the brain. Nature 459, 1005–1009 19421193 10.1038/nature08021 PMC2698953

[b7-0070363] Briggs K. Al Mahrouki A. Nofiele J. El-Falou A. Stanisz M. Kim H. C. Kolios M. C. Czarnota G. J. (2013). Non-invasive monitoring of ultrasound-stimulated microbubble radiation enhancement using photoacoustic imaging. Technol. Cancer Res. Treat. [Epub ahead of print] DOI: 10.7785/tcrtexpress.2013.600266 PMC4527466 24000993

[b8-0070363] Butterworth K. T. McCarthy H. O. Devlin A. Ming L. Robson T. McKeown S. R. Worthington J. (2008). Hypoxia selects for androgen independent LNCaP cells with a more malignant geno- and phenotype. Int. J. Cancer 123, 760–768 18512241 10.1002/ijc.23418

[b9-0070363] Cébe-Suarez S. Zehnder-Fjällman A. Ballmer-Hofer K. (2006). The role of VEGF receptors in angiogenesis; complex partnerships. Cell. Mol. Life Sci. 63, 601–615 16465447 10.1007/s00018-005-5426-3 PMC2773843

[b10-0070363] Cervi D. Shaked Y. Haeri M. Usenko T. Lee C. R. Haigh J. J. Nagy A. Kerbel R. S. Yefenof E. Ben-David Y. (2007). Enhanced natural-killer cell and erythropoietic activities in VEGF-A-overexpressing mice delay F-MuLV-induced erythroleukemia. Blood 109, 2139–2146 17053052 10.1182/blood-2005-11-026823 PMC1801043

[b11-0070363] Connell P. P. Hellman S. (2009). Advances in radiotherapy and implications for the next century: a historical perspective. Cancer Res. 69, 383–392 19147546 10.1158/0008-5472.CAN-07-6871

[b12-0070363] Cosgrove D. (2006). Ultrasound contrast agents: an overview. Eur. J. Radiol. 60, 324–330 16938418 10.1016/j.ejrad.2006.06.022

[b13-0070363] Czarnota C. J. Karshafian R. Burns P. N. Wong S. Al-Mahrouki A. Lee J. W. Caissie A. Tran W. Kim C. Furukawa M. (2012). Tumor radiation response enhancement by acoustical stimulation of the vasculature. Proc. Natl. Acad. Sci. 109, 11904–11905 10.1073/pnas.1200053109 PMC3409730 22778441

[b14-0070363] Diehn M. Cho R. W. Lobo N. A. Kalisky T. Dorie M. J. Kulp A. N. Qian D. Lam J. S. Ailles L. E. Wong M. (2009). Association of reactive oxygen species levels and radioresistance in cancer stem cells. Nature 458, 780–783 19194462 10.1038/nature07733 PMC2778612

[b15-0070363] Diepart C. Karroum O. Magat J. Feron O. Verrax J. Calderon P. B. Grégoire V. Leveque P. Stockis J. Dauguet N. (2012). Arsenic trioxide treatment decreases the oxygen consumption rate of tumor cells and radiosensitizes solid tumors. Cancer Res. 72, 482–490 22139377 10.1158/0008-5472.CAN-11-1755

[b16-0070363] Dow L. W. Bhakta M. Wilimas J. (1982). Clonogenic assay for Wilms’ tumor: improved technique for obtaining single-cell suspensions and evidence for tumor cell specificity. Cancer Res. 42, 5262–5264 6291751

[b17-0070363] Ebos J. M. Lee C. R. Cruz-Munoz W. Bjarnason G. A. Christensen J. G. Kerbel R. S. (2009). Accelerated metastasis after short-term treatment with a potent inhibitor of tumor angiogenesis. Cancer Cell 15, 232–239 19249681 10.1016/j.ccr.2009.01.021 PMC4540346

[b18-0070363] Folkman J. Camphausen K. (2001). Cancer. What does radiotherapy do to endothelial cells? Science 293, 227–228 11452105 10.1126/science.1062892

[b19-0070363] Garcia-Barros M. Paris F. Cordon-Cardo C. Lyden D. Rafii S. Haimovitz-Friedman A. Fuks Z. Kolesnick R. (2003). Tumor response to radiotherapy regulated by endothelial cell apoptosis. Science 300, 1155–1159 12750523 10.1126/science.1082504

[b20-0070363] Garden A. S. Maor M. H. Yung W. K. Bruner J. M. Woo S. Y. Moser R. P. Lee Y. Y. (1991). Outcome and patterns of failure following limited-volume irradiation for malignant astrocytomas. Radiother. Oncol. 20, 99–110 1851573 10.1016/0167-8140(91)90143-5

[b21-0070363] Goertz D. E. Todorova M. Mortazavi O. Agache V. Chen B. Karshafian R. Hynynen K. (2012). Antitumor effects of combining docetaxel (taxotere) with the antivascular action of ultrasound stimulated microbubbles. PLoS ONE 7, e52307 23284980 10.1371/journal.pone.0052307 PMC3527530

[b22-0070363] Gravdal K. Halvorsen O. J. Haukaas S. A. Akslen L. A. (2009). Proliferation of immature tumor vessels is a novel marker of clinical progression in prostate cancer. Cancer Res. 69, 4708–4715 19487287 10.1158/0008-5472.CAN-08-4417

[b23-0070363] Haag P. Frauscher F. Gradl J. Seitz A. Schäfer G. Lindner J. R. Klibanov A. L. Bartsch G. Klocker H. Eder I. E. (2006). Microbubble-enhanced ultrasound to deliver an antisense oligodeoxynucleotide targeting the human androgen receptor into prostate tumours. J. Steroid Biochem. Mol. Biol. 102, 103–113 17055720 10.1016/j.jsbmb.2006.09.027

[b24-0070363] Hernot S. Klibanov A. L. (2008). Microbubbles in ultrasound-triggered drug and gene delivery. Adv. Drug Deliv. Rev. 60, 1153–1166 18486268 10.1016/j.addr.2008.03.005 PMC2720159

[b25-0070363] Huang S. Ingber D. E. (1999). The structural and mechanical complexity of cell-growth control. Nat. Cell Biol. 1, E131–E138 10559956 10.1038/13043

[b26-0070363] Huang Z. Mayr N. A. Yuh W. T. Lo S. S. Montebello J. F. Grecula J. C. Lu L. Li K. Zhang H. Gupta N. (2010). Predicting outcomes in cervical cancer: a kinetic model of tumor regression during radiation therapy. Cancer Res. 70, 463–470 20068180 10.1158/0008-5472.CAN-09-2501 PMC2822442

[b27-0070363] Ibsen S. Schutt C. E. Esener S. (2013). Microbubble-mediated ultrasound therapy: a review of its potential in cancer treatment. Drug Des Devel Ther 7, 375–388 10.2147/DDDT.S31564 PMC3650568 23667309

[b28-0070363] Jokilehto T. Rantanen K. Luukkaa M. Heikkinen P. Grenman R. Minn H. Kronqvist P. Jaakkola P. M. (2006). Overexpression and nuclear translocation of hypoxia-inducible factor prolyl hydroxylase PHD2 in head and neck squamous cell carcinoma is associated with tumor aggressiveness. Clin. Cancer Res. 12, 1080–1087 16489060 10.1158/1078-0432.CCR-05-2022

[b29-0070363] Kamat A. A. Merritt W. M. Coffey D. Lin Y. G. Patel P. R. Broaddus R. Nugent E. Han L. Y. Landen C. N. Jr Spannuth W. A. (2007). Clinical and biological significance of vascular endothelial growth factor in endometrial cancer. Clin. Cancer Res. 13, 7487–7495 18094433 10.1158/1078-0432.CCR-07-1017

[b30-0070363] Karshafian R. Bevan P. D. Williams R. Samac S. Burns P. N. (2009). Sonoporation by ultrasound-activated microbubble contrast agents: effect of acoustic exposure parameters on cell membrane permeability and cell viability. Ultrasound Med. Biol. 35, 847–860 19110370 10.1016/j.ultrasmedbio.2008.10.013

[b31-0070363] Kee N. Sivalingam S. Boonstra R. Wojtowicz J. M. (2002). The utility of Ki-67 and BrdU as proliferative markers of adult neurogenesis. J. Neurosci. Methods 115, 97–105 11897369 10.1016/s0165-0270(02)00007-9

[b32-0070363] Kim H.C. Al-Mahrouki A. Gorjizadeh A. Karshafian R. Czarnota G.J. (2013). Effects of biophysical parameters in enhancing radiation responses of prostate tumors with ultrasound-stimulated microbubbles. Ultrasound Med. Biol. 39, 1376–1387 23643061 10.1016/j.ultrasmedbio.2013.01.012

[b33-0070363] Korpanty G. Carbon J. G. Grayburn P. A. Fleming J. B. Brekken R. A. (2007). Monitoring response to anticancer therapy by targeting microbubbles to tumor vasculature. Clin. Cancer Res. 13, 323–330 17200371 10.1158/1078-0432.CCR-06-1313

[b34-0070363] Leung T. H. Ngan H. Y. (2010). Interaction of TAp73 and breast cancer-associated gene 3 enhances the sensitivity of cervical cancer cells in response to irradiation-induced apoptosis. Cancer Res. 70, 6486–6496 20647320 10.1158/0008-5472.CAN-10-0688

[b35-0070363] Lukas J. Lukas C. Bartek J. (2011). More than just a focus: The chromatin response to DNA damage and its role in genome integrity maintenance. Nat. Cell Biol. 13, 1161–1169 21968989 10.1038/ncb2344

[b36-0070363] Mammoto A. Connor K. M. Mammoto T. Yung C. W. Huh D. Aderman C. M. Mostoslavsky G. Smith L. E. Ingber D. E. (2009). A mechanosensitive transcriptional mechanism that controls angiogenesis. Nature 457, 1103–1108 19242469 10.1038/nature07765 PMC2708674

[b37-0070363] Mantovani A. Allavena P. Sica A. Balkwill F. (2008). Cancer-related inflammation. Nature 454, 436–444 18650914 10.1038/nature07205

[b38-0070363] Matthews B. D. Overby D. R. Mannix R. Ingber D. E. (2006). Cellular adaptation to mechanical stress: role of integrins, Rho, cytoskeletal tension and mechanosensitive ion channels. J. Cell Sci. 119, 508–518 16443749 10.1242/jcs.02760

[b39-0070363] Mazzone M. Dettori D. Leite de Oliveira R. Loges S. Schmidt T. Jonckx B. Tian Y. M. Lanahan A. A. Pollard P. Ruiz de Almodovar C. (2009). Heterozygous deficiency of PHD2 restores tumor oxygenation and inhibits metastasis via endothelial normalization. Cell 136, 839–851 19217150 10.1016/j.cell.2009.01.020 PMC4037868

[b40-0070363] Miller D. L. Dou C. Sorenson D. Liu M. (2011). Histological observation of islet hemorrhage induced by diagnostic ultrasound with contrast agent in rat pancreas. PLoS ONE 6, e21617 21738734 10.1371/journal.pone.0021617 PMC3125214

[b41-0070363] Nofiele J. T. Karshafian R. Furukawa M. Al Mahrouki A. Giles A. Wong S. Czarnota G. J. (2013). Ultrasound-activated microbubble cancer therapy: ceramide production leading to enhanced radiation effect in vitro. Technol. Cancer Res. Treat. 12, 53–60 22905807 10.7785/tcrt.2012.500253 PMC4527482

[b42-0070363] Pàez-Ribes M. Allen E. Hudock J. Takeda T. Okuyama H. Viñals F. Inoue M. Bergers G. Hanahan D. Casanovas O. (2009). Antiangiogenic therapy elicits malignant progression of tumors to increased local invasion and distant metastasis. Cancer Cell 15, 220–231 19249680 10.1016/j.ccr.2009.01.027 PMC2874829

[b43-0070363] Paris F. Fuks Z. Kang A. Capodieci P. Juan G. Ehleiter D. Haimovitz-Friedman A. Cordon-Cardo C. Kolesnick R. (2001). Endothelial apoptosis as the primary lesion initiating intestinal radiation damage in mice. Science 293, 293–297 11452123 10.1126/science.1060191

[b44-0070363] Pchejetski D. Bohler T. Brizuela L. Sauer L. Doumerc N. Golzio M. Salunkhe V. Teissié J. Malavaud B. Waxman J. (2010). FTY720 (fingolimod) sensitizes prostate cancer cells to radiotherapy by inhibition of sphingosine kinase-1. Cancer Res. 70, 8651–8661 20959468 10.1158/0008-5472.CAN-10-1388

[b45-0070363] Phillips L. C. Klibanov A. L. Wamhoff B. R. Hossack J. A. (2010). Targeted gene transfection from microbubbles into vascular smooth muscle cells using focused, ultrasound-mediated delivery. Ultrasound Med. Biol. 36, 1470–1480 20800174 10.1016/j.ultrasmedbio.2010.06.010 PMC2930891

[b46-0070363] Reynolds A. R. Hart I. R. Watson A. R. Welti J. C. Silva R. G. Robinson S. D. Da Violante G. Gourlaouen M. Salih M. Jones M. C. (2009). Stimulation of tumor growth and angiogenesis by low concentrations of RGD-mimetic integrin inhibitors. Nat. Med. 15, 392–400 19305413 10.1038/nm.1941

[b47-0070363] Spiegelhalter D. Pearson M. Short I. (2011). Visualizing uncertainty about the future. Science 333, 1393–1400 21903802 10.1126/science.1191181

[b48-0070363] Tran W. T. Iradji S. Sofroni E. Giles A. Eddy D. Czarnota G. J. (2012). Microbubble and ultrasound radioenhancement of bladder cancer. Br. J. Cancer 107, 469–476 22790798 10.1038/bjc.2012.279 PMC3405216

[b49-0070363] Wei D. Li H. Yu J. Sebolt J. T. Zhao L. Lawrence T. S. Smith P. G. Morgan M. A. Sun Y. (2012). Radiosensitization of human pancreatic cancer cells by MLN4924, an investigational NEDD8-activating enzyme inhibitor. Cancer Res. 72, 282–293 22072567 10.1158/0008-5472.CAN-11-2866 PMC3251739

[b50-0070363] Wijsman J. H. Jonker R. R. Keijzer R. van de Velde C. J. Cornelisse C. J. van Dierendonck J. H. (1993). A new method to detect apoptosis in paraffin sections: in situ end-labeling of fragmented DNA. J. Histochem. Cytochem. 41, 7–12 7678025 10.1177/41.1.7678025

[b51-0070363] Zlobec I. Höller S. Tornillo L. Terracciano L. Lugli A. (2009). Combined histomorphologic and immunohistochemical phenotype to predict the presence of vascular invasion in colon cancer. Dis. Colon Rectum 52, 1114–1121 19581855 10.1007/DCR.0b013e31819eefd9

