# Supplementary material for: Cellular characterization of ultrasound-stimulated microbubble radiation enhancement in a prostate cancer xenograft model
Source: Dis Model Mech. 2014 Jan 30;7(3):363–72. doi: 10.1242/dmm.012922 (PMC3944496; doi:10.1242/dmm.012922)
Supplement: Supplementary Material [file supp_7_3_363__index.html]

Cellular characterization of ultrasound-stimulated microbubble radiation enhancement in a prostate cancer xenograft model — Supplementary Material 

# Cellular characterization of ultrasound-stimulated microbubble radiation enhancement in a prostate cancer xenograft model

## DMM012922 Supplementary Material

**Files in this Data Supplement:**

- **Supplementary Material**
