# Supplementary material for: Cellular characterization of ultrasound-stimulated microbubble radiation enhancement in a prostate cancer xenograft model
Source: Dis Model Mech. 2014 Jan 30;7(3):363–72. doi: 10.1242/dmm.012922 (PMC3944496; doi:10.1242/dmm.012922)
Supplement: Supplementary Material [file supp_7.3.363_DMM012922.pdf]

Supplementary Information

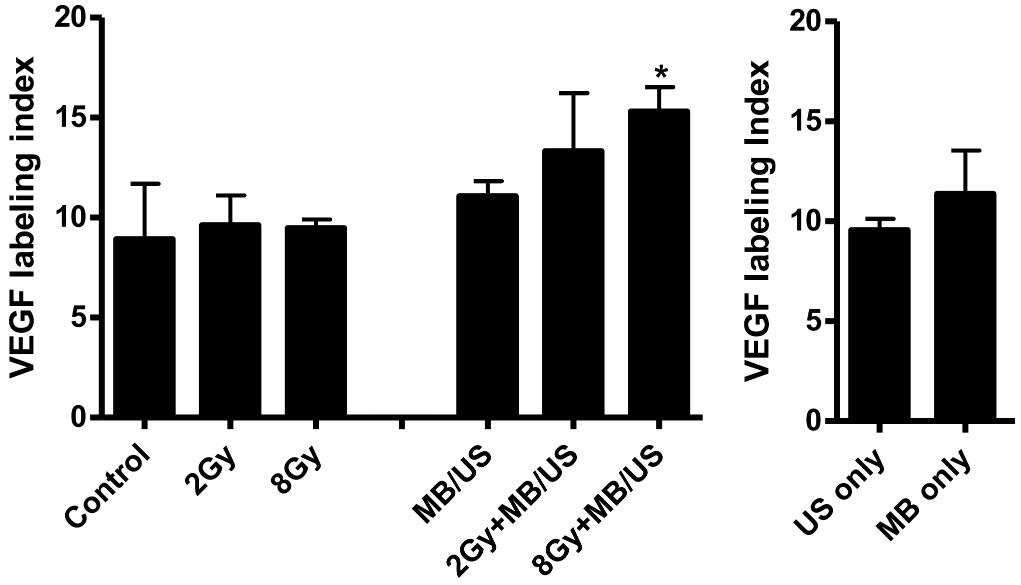

<u>**Figure S1**</u>

Controls treated with ultrasound only (no bubbles) or bubbles only (no ultrasound) were not significantly different from the controls that were not treated.

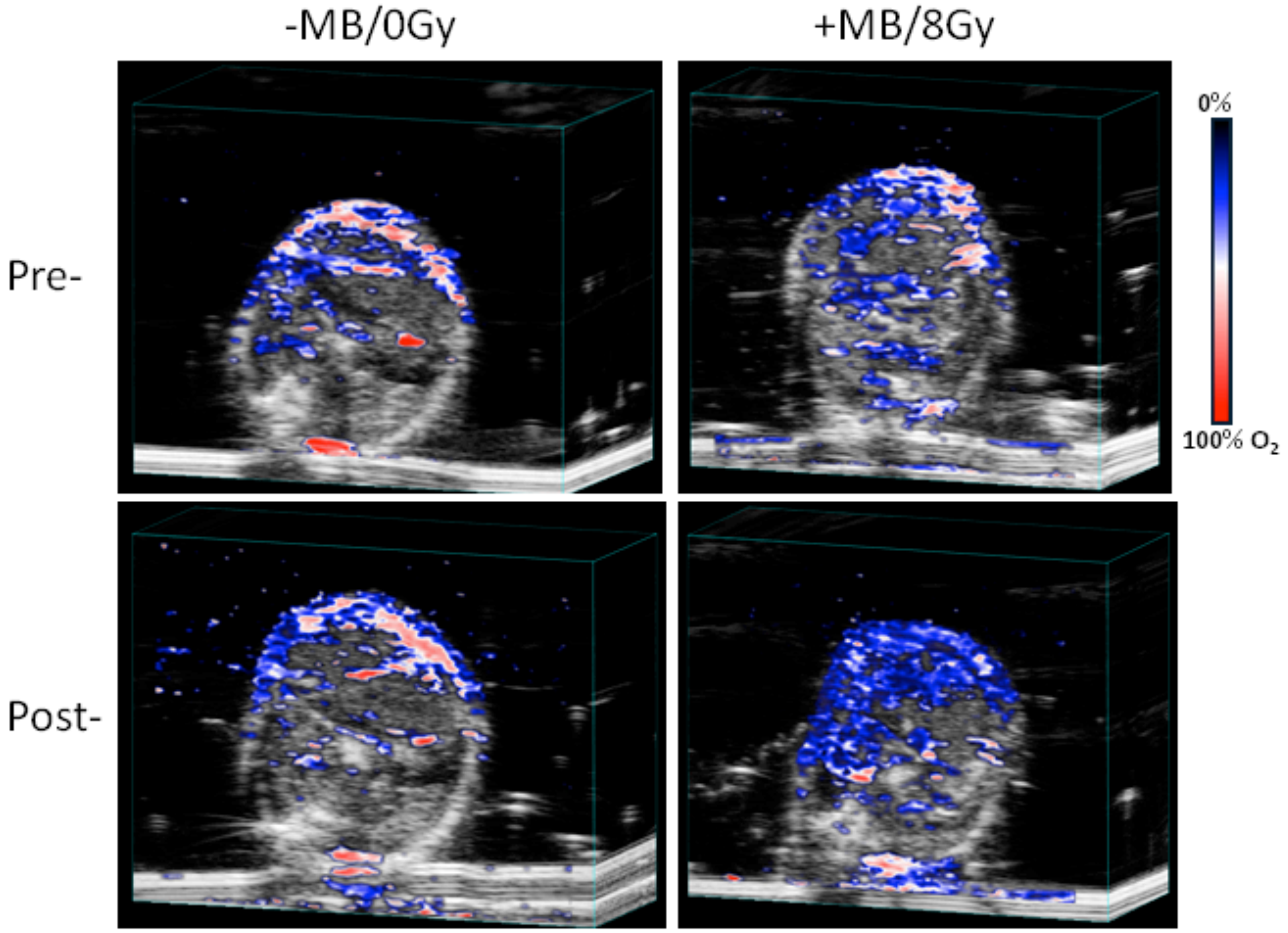

Figure S2

Photoacoustics images of oxygen saturation in PC3 xenograft tumors.

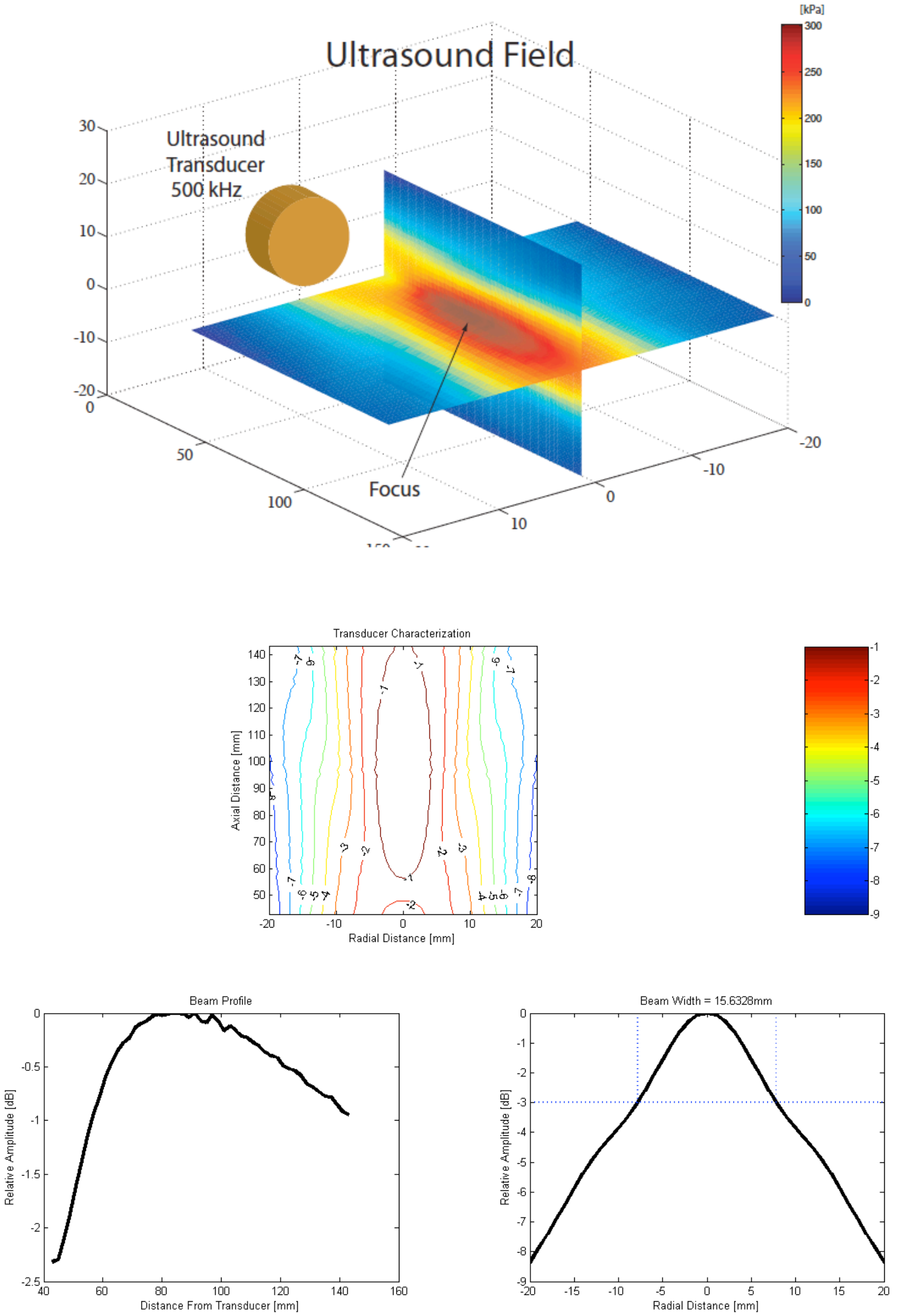

Figure S3

Characterization maps of the ultrasound transducer used in this study.
